# An inactivated gE-deleted pseudorabies vaccine provides complete clinical protection and reduces virus shedding against challenge by a Chinese pseudorabies variant

**DOI:** 10.1186/s12917-016-0897-z

**Published:** 2016-12-07

**Authors:** Jichun Wang, Rongli Guo, Yongfeng Qiao, Mengwei Xu, Zhisheng Wang, Yamei Liu, Yiqi Gu, Chang Liu, Jibo Hou

**Affiliations:** 1 National Research Center of Engineering and Technology for Veterinary Biologicals/Institute of Veterinary Medicine, Jiangsu Academy of Agricultural Sciences, Nanjing, Jiangsu 210014 China; 2 College of Veterinary Medicine, Nanjing Agricultural University, Nanjing, 210095 China; 3 Jiangsu Co-innovation Center for Prevention and Control of Important Animal Infectious Diseases and Zoonoses, Yangzhou, China

**Keywords:** Pseudorabies virus emerging variant, gE deletion, Inactivated vaccine, Adjuvant, Bacterial artificial chromosome, Challenge protection

## Abstract

**Background:**

Since the end of 2011 an outbreak of pseudorabies affected Chinese pig herds that had been vaccinated with the commercial vaccine made of Bartha K61 strain. It is now clear that the outbreak was caused by an emergent PRV variant. Even though vaccines made of PRV Bartha K61 strain can confer certain cross protection against PRV variants based on experimental data, less than optimal clinical protection and virus shedding reduction were observed, making the control or eradication of this disease difficult.

**Results:**

An infectious clone of PRV AH02LA strain was constructed to generate a gE deletion mutant PRV(LA-A^B^) strain. PRV(LA-A^B^) strain can reach a titer of 10^8.43^ TCID_50_ /mL (50% tissue culture infectious dose) on BHK-21 cells. To evaluate the efficiency of the inactivated vaccine made of PRV(LA-A^B^) strain, thirty 3-week-old PRV-negative piglets were divided randomly into six groups for vaccination and challenge test. All five piglets in the challenge control showed typical clinical symptoms of pseudorabies post challenge. Sneezing and nasal discharge were observed in four and three piglets in groups C(vaccinated with inactivated PRV Bartha K61 strain vaccine) and D(vaccinated with live PRV Bartha K61 strain vaccine) respectively. In contrast, piglets in both groups A(vaccinated with inactivated PRV LA-AB strain vaccine) and B(vaccinated with inactivated PRV LA-A^B^ strain vaccine with adjuvant) presented mild or no clinical symptoms. Moreover, viral titers detected via nasal swabs were approximately 100 times lower in group B than in the challenge control, and the duration of virus shedding (3–4 days) was shorter than in either the challenge control (5–10 days) or groups C and D (5–6 days).

**Conclusions:**

The infectious clone constructed in this study harbors the whole genome of the PRV variant AH02LA strain. The gE deletion mutant PRV(LA-A^B^)strain generated from PRV AH02LA strain can reach a high titer on BHK-21 cells. An inactivated vaccine of PRV LA-A^B^ provides clinical protection and significantly reduces virus shedding post challenge, especially if accompanied by the adjuvant CVC1302. While Inactivated or live vaccines made of PRV Barth K61 strain can provide only partial protection in this test.

## Background

Pseudorabies, caused by pseudorabies virus(PRV), has leaded to significant economic losses to the pig industry in many countries. Pseudorabies is a porcine infectious disease that can be effectively controlled using gene deletion marked vaccines and serum monitoring, enabling differentiation of vaccinated from infected animals [1–3]. However, in 2011 an outbreak of pseudorabies affected Chinese pig herds that had been vaccinated with the standard PRV Bartha K61 strain [4–9]. It is now clear that the outbreak was caused by an emergent PRV variant [5–7, 9–13]. As a support for this finding, the widely used PRV Bartha K61 strain was shown to be incapable of providing complete protection against this new PRV variant in several experimental studies [4–8]. Thus, a more efficacious vaccine is perceived as a necessary tool to help affected herds revert to PRV virus negative status.

To develop an effective vaccine, gene deletion mutants were generated from the new PRV strain and evaluated for protection efficacy. Although live vaccine candidates based on gE, gE/gI, or TK/gE/gI gene deletion mutants are assumed to be highly protective, the safety issues of these preparations have to be demonstrated by lengthy testing [4, 14–18]. Alternatively, inactivated version of vaccine from the same mutants are promising to provide clinical protection as well based on relevant data about inactivated Bartha strain.

In this study, LA-A^B^, a gE deleted mutant of the emerging PRV strain AH02LA, was constructed using a bacterial artificial chromosome clone. A vaccine utilizing this mutant was evaluated with and without adjuvant in regard to its ability to provide clinical protection and reduce virus shedding after lethal challenge.

## Methods

### Cells and viruses

BHK-21 cells (CVCC:CL5, from China Veterinary Culture Collection Center) and chicken embryo cells (CECs) (SPF chicken embryo eggs were from Beijing Merial Vital Laboratory Animal Technology Co., Ltd) were prepared using standard methods. The PRV Bartha K61 strain was kindly provided by Professor Ping Jiang of Nanjing Agricultural University. Virus cultures and stocks were prepared using CECs or BHK-21 cells before at -70 °C. Virus titers were determined by TCID_50_ on BHK-21 cells following a standard protocol. Viral DNA extraction and transfection of plasmid, virus or bacterial artificial chromosome(BAC) DNAs were performed as described previously [19, 20].

### Plasmid and BAC manipulation

The mini-F transfer vector pHA2-pUC19-H1-H2 was constructed as described [16]. Plasmid and BAC DNAs were isolated using commercial kits (Invitrogen PureLink® Quick Plasmid Miniprep and Qiagen Large-Construct Kit) following the manufacturer’s instructions. Next, restriction fragment length polymorphism(RFLP) analyses of PRV BACs was conducted using the restriction endonucleases *BamH* I, *Kpn* I, *Pst* I and *Sph* I and digestion conditions recommended by the supplier (Takara). Electrocompetent *E.coli* cells (DH10B) were obtained commercially (Invitrogen). *E.coli* GS1783 was made electrocompetent using published protocols [21–23]. Electroporation was performed to transform viral or BAC DNA following established methods [23–25].

### PCR and sequencing

**Fig. 1:**
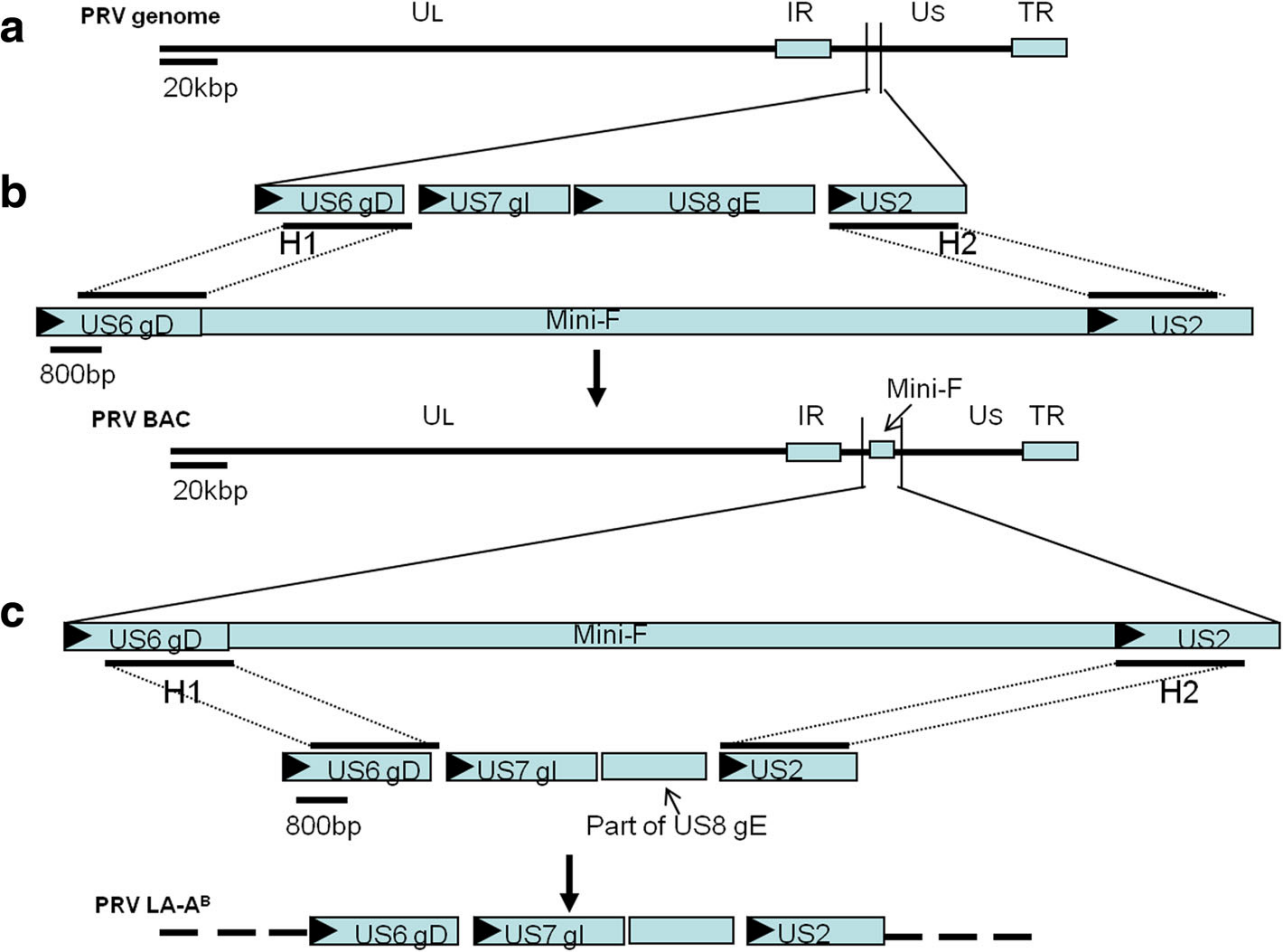
Construction of mini-F recombinant PRV AH02LA strain and its gE deletion mutant virus (LA-A^B^). **a** The whole genome of PRV. **b** Homologous recombination was conducted to insert mini-F in lieu of gI and gE to generate the mini-F recombinant PRV AH02LA strain for BAC construction. **c** Another recombination was performed to recover the whole gI gene and part of gE gene, the resulting BAC^PRV-G^ was used to generate the gE deletion LA-A^B^ strain. Scales in bp or kbp are provided

A pair of primers (Table 1) was designed from reference sequence (GenBank:NC_006151.1) to amplify sequences of gI gene with isolated PRV AH02LA strain DNA as template. These primers were also used for sequencing gI gene (GeneScript, Nanjing China). To obtain the gE deletion, overlap PCR was conducted with two pairs of primers, ΔgE T^V^ overlap1 and ΔgE T^V^ overlap2 (Table 1). Briefly, with PRV AH02LA DNA as template, two PCR reactions were performed separately with the above two primer pairs. Next, the PCR products were gel purified to serve as templates in another PCR reaction with primers ΔgE T^V^ overlap1 F and ΔgE T^V^ overlap2 R to produce a fragment for gE deletion mutant generation. Thus the generated mutant, consists of upstream sequences, the intact gI gene, part of gE gene(1299 bp to 1735 bp of gE ORF), and downstream sequences (Fig. 1). Finally, the primers PRV Homo-1 F and PRV Homo-2R [16] were used to amplify the repairing viral DNA with the PRV AH02LA DNA as template.

**Table 1:** Primers for PCR or sequencing

Primer	Sequence
PRV gD F	5’-AACACCTAATTTGCGTACGGC-3’
PRV gD R	5’-TCATCATCGACGCCGGTACT-3’
PRV gI F	5’-TGGGCGTGTGCGTCTACATCT-3’
PRV gI R	5’-CAACCCCGGTGTGTGTGAGA-3’
ΔgE T^V^ overlap1 F	5’-CCACGCCCAGCGGTCCATAAAATTGGGT-3’
ΔgE T^V^ overlap1 R	5’- TCATCACGAAGGAGCCCAGCAAAGGGCCGCATGG TCTCAACC-3’
ΔgE T^V^ overlap2 F	5’-GCTGGGCTCCTTCGTGATGA-3’
ΔgE T^V^ overlap2 R	5’-TCACGATCTGGGCATGCAGG-3’
SEQ-gI/gE F 1	5’-TTGCGTACGGCCTTGCTTAC-3’
SEQ-gI/gE F 2	5’-GACTACATGTTCCCCACGGA-3’
SEQ-gI/gE F 3	5’-ACGCCGTACGCCATCGACCC-3’
SEQ-gI/gE F 4	5’-ACGCTGCTGTTTCTGGAGGG-3’
SEQ-gI/gE F 5	5’-ACGAAGAGGAGGAGGACGAG-3’
SEQ-gI/gE F 6	5’-ACCATGCGGCCCTTTCTGCT-3’
SEQ-gI/gE F 7	5’-ACCACGGTGTGCTTCGAGAC-3’
SEQ-gI/gE F 8	5’-TGTACGAGCCCTGCATCTACCACCC-3’
SEQ-gI/gE F 9	5’-ACTACTACGACGGCGACGACGACGA-3’
SEQ-gI/gE F 10	5’-AACGAGACGCCCAGCGAGTT-3’
SEQ-gI/gE F 11	5’-AAGGTGCTCACCGAGTGGTGCTACG-3’
SEQ-gI/gE R 1	5’-TCTAGGAGATGGTACATCGCGGGGC-3’
SEQ-gI/gE R 2	5’-TGGTGATGTAGAACGGCGCC-3’
SEQ-gI/gE R 3	5’-ACAGCGAGCAGATGACCAGC-3’
SEQ-gI/gE R 4	5’-TCGCTGCTGAACTCGTCCTC-3’
SEQ-gI/gE R 5	5’-ATCACGAGCACGTACAGCCC-3’
SEQ-gI/gE R 6	5’-TGTAGAGGCCCGTGTCGTTG-3’
SEQ-gI/gE R 7	5’-AAAGGGCCGCATGGTCTCAACC-3’
SEQ-gI/gE R 8	5’-TCCTCCTCCTCTTCGTCGGA-3’
SEQ-gI/gE R 9	5’-AAAGAGGTCCGTGGTCCCGTTCAC-3’
SEQ-gI/gE R 10	5’-AGATGTAGACGCACACGCCCACCAG-3’
SEQ-gI/gE R 11	5’-GGGAACATGTAGTCCGCGGA-3’

### Multistep growth kinetics

Multistep growth kinetics of parental AH02LA, gE deletion LA-A^B^, and repair AH02LA^R^ viruses were tested on BHK-21 cells following methods as described [26] on monolayers (1 × 10^6^ cells) of BHK-21 cells with a multiplicity of infection(MOI)of 0.01. Three independent experiments were conducted and one-way ANOVA (SPSS software package) was employed to identify significant differences.

### LD_50_ determination for PRV AH02LA strain

To determine the LD_50_ of the virulent PRV AH02LA strain, the virus (TCID_50_ = 10^-8.25^/mL) was serially diluted with DMEM to 10^-1^, 10^-2^, 10^-3^ and 10^-4^. Twenty-five 77-day-old piglets were randomly divided into five groups. Piglets in each challenge group were inoculated intranasally(I.N.) with 1 mL diluted virus of different titers. Animals were observed for additional fourteen days. The number of piglets that died in each group was recorded to calculate LD_50_ using the Karber method.

### Construction of PRV BAC and deletion mutants

The PRV BAC was constructed from an isolate of the emergent PRV variant following published methods (Fig. 1) [16, 26]. Briefly, After cotransfection of ~1.5 μg PRV AH02LA strain DNA and ~4 μg pHA2-pUC19-H1-H2 DNA, mini-F recombinant viruses with green fluorescence were purified to obtain a homogeneous population [27]. Next, mini-F containing PRV DNA was isolated and then transferred into *E. coli* cells DH10B and subsequently into *E.coli* strain GS1783 cells by electroporation for mutagenesis [23]. Finally, BAC DNA was isolated and RFLPs were identified as described [24] using *BamH* I, *Kpn* I, *Pst* I and *Sph* I, using the complete genome sequence of PRV ZJ01 strain (GenBank: KM061380.1) as a reference for *in silico* prediction.

To generate the gE deletion mutant of PRV AH02LA strain, another homologous recombination was performed to recover the gI gene and the undeleted sequences of gE as shown in Fig. 1. Briefly, PCR with primers ΔgE T^V^ overlap1 F and ΔgE T^V^ overlap2 R (Table 1) was performed with the PRV AH02LA DNA as template. And then CECs were co-transfected with approximately 5 μg PRV BAC DNA and 2 μg DNA of fragment from the second PCR reaction of overlap PCR with primersΔgE T^V^ overlap1 F andΔgE T^V^ overlap2 R by calcium phosphate precipitation. Using illumination at 488 nm, non-fluorescent virus isolates were selected and purified to homogeneity. Product structure was confirmed by sequencing with the same primers and other specific primers from the SEQ-gI/gE series (Table 1).

For generation of the gI/gE repair virus via homologous recombination, CECs were transfected with approximately 5 μg PRV BAC DNA and 2 μg PCR product generated using DNA of parental PRV as template and primers matching PRV Homo 1 F and PRV Homo 2R [16] (Fig. 1). Two to three days after transfection, non-fluorescent plaques (488 nm) were isolated and purified to obtain a homogeneous population. The structure of the recovered viruses was confirmed by PCR and sequencing with primers PRV Homo 1 F, PRV Homo 2R and sequencing primers of SEQ-gI/gE (Table 1).

### Preparation of vaccine

PRV LA-A^B^ and Bartha K61 viruses were propagated in BHK-21 cells using a 5 liter bioreactor, each virus stock was diluted to 10^8.5^ TCID_50_/mL. After inactivation with formalin (Sigma-Aldrich) as described [16], vaccines were prepared using mineral oil adjuvant (1:3 water in oil). All vaccines were confirmed to be free of bacteria and fungi, and had the expected physical properties according to standard protocols. Vaccines were stored at 4 °C until use.

### Test of vaccine efficacy

Thirty piglets of 21-day-old (from Zhengzhuquan Pig Breeding Farm in Pukou district, Nanjing, China) were randomly divided into 6 groups (A-F) and housed in separated facilities. All piglets were antibody negative for PRV gB and gE, and free of porcine reproductive and respiratory syndrome virus, porcine parvovirus, porcine circovirus 2 and classical swine fever virus. All inoculations were given intramuscularly(I.M.) at 2 mL/pig. Group A piglets were inoculated with LA-A^B^, group B piglets with LA-A^B^ plus adjuvant CVC1302 (Patent: CN103083663B, kindly provided by the group of Dr. Qisheng Zheng in our institute), group C piglets with inactivated Bartha K61, group D piglets with live Bartha K61 of 1 × 10^5.0^ TCID_50_ virus/dose, and groups E and F piglets were dosed with PBS only. Piglets in groups A, B, and C were inoculated at 28 days of age and again at 56 days. Group D piglets were inoculated once, at 28-day-old. Group E and F piglets were dosed once, at 28 days of age. Three weeks after the second inoculation (at which time the piglets were 11-week-old), groups A, B, C, D and E were challenged I.N. with 3 × LD_50_ PRV AH02LA per piglet. Group F piglets were not challenged, serving as a negative control. Serum samples from all piglets were collected before inoculation, and at 7, 14, 21, 28, 35, 42 and 49 days post inoculation, and again at 14 days post challenge(P.C.). Serum was subjected to ELISA tests for PRV gB and gE antibodies, and cross-neutralizing (NA) antibodies against AH02LA or Bartha K61. Body temperature, clinical signs, and virus shedding were monitored and recorded daily until 14 days P.C.. The presence of lung lesions for died or survived piglets was noted at the end of the test.

### Test for serological antibodies

ELISA tests were performed with the PRV gE or gB antibody detection kit (IDEXX, Maine, USA) following manufacturer’s instructions. Cross neutralization tests were conducted according to the standard methods published by OIE (Veterinary Pharmacopoeia of the People's Republic of China (2010)) with slight modification. Briefly, serum samples were diluted two-fold with PBS, mixed with 100 TCID_50_ PRV AH02LA or PRV Bartha K61 virus, and incubated for 1 h at 37 °C. The serum-virus mixture was used to inoculate BHK-21 cells which were then incubated at 37 °C, 5% CO_2_ for 3–4 days to observe the development of CPE. Titers of neutralizing antibody were expressed as nlog_2_ of highest dilution at which no CPE was observed, and mean titers of each group were calculated for comparison.

### Detection of virus shedding

Nasal swab samples were collected from all piglets before challenge and daily to 14 days after challenge. After shaking at 5000–8000 RPM for 1–2 min, one freeze-thaw cycle (-70 °C and 37 °C) was conducted to release viruses from nasal swabs. Samples were centrifuged at 10,000 RPM for 15 min to pellet tissue and cell debris, the supernatants were collected to determine virus titers. Viruses titers were expressed as TCID_50_ following the method of Karber.

All animal studies were conducted following guidelines provided by the Institutional Biosafety Committee and approved by the Institutional Animal Care and Use Committee at the Jiangsu Academy of Agriculture Sciences. Experiments involving virulent PRV were conducted under Biosafety Level 2+ containment.

## Results

### Isolation and identification of an emergent pseudorabies virus variant (AH02LA strain)

BHK-21 cells, inoculated with a sample of brain tissue from a stillborn piglet, exhibited cytopathogenic effects (CPE) after 3 days. After three rounds of plaque purification, a homogeneous population of virus was isolated and designated as the AH02LA strain. Using the viral DNA as template and the primers PRV gI F/ R (Table 1), PCR generated a product of 1250–1400 bp. Sequencing established that this fragment was homologous to the PRV gI gene. The AH02LA strain can be specifically neutralized with the standard antibodies against PRV (CIVDC, Beijing China) with a neutralizing index of 10,000. These results demonstrate that a variant PRV field isolate was identified.

### Pathology of the PRV AH02LA strain

The LD_50_ of PRV AH02LA (Batch: JJ-R5) was determined using 77-day-old piglets free of antibodies against PRV. Three of five piglets in the 10^-5^ dosage group, and all of the piglets in the 10^-4^ dosage group, showed typical symptoms of PRV infection: sneezing, purulent nasal discharge, difficulty in breathing and ataxia, indicating the high virulence of this strain. The LD_50_ was 10^-2.32^/mL. The highly virulent character of AH02LA is shared by other emergent PRV strains isolated during the 2011 outbreaks [4–8]. The significant difference between these emerging strains and the conventional strain S (HWBD, Harbin China) is that the former cause mortalities in not only young piglets but also much older pigs, whereas the conventional strain S causes death only in young (less than 15-day-old) piglets (Veterinary Pharmacopoeia of the People's Republic of China (2005)).

### Construction of a bacterial artificial chromosome containing the AH02LA genome

**Fig. 2:**
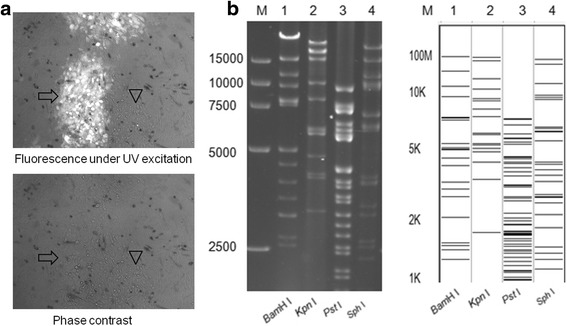
Plaques of mini-F recombinant PRV AH02LA strain and RFLP of BAC^PRV-G^. **a** Images of mini-F recombinant PRV AH02LA and the parental AH02LA plaques under UV excitation (upper) and phase contrast (lower) are shown. Arrowhead shows a plaque of parental PRV AH02LA virus and arrow shows a plaque formed by mini-F recombinant PRV AH02LA. Each panel represents a view of 200 × 200 μm in size. **b** RFLP of BAC^PRV-G^, DNA from PRV AH02LA BAC clone BAC^PRV-G^ was prepared by mini-prep and digested with *BamH* I, *Kpn* I, *Pst* I and *Sph* I (lanes 1–4). The digests were separated by 0.8% agarose gel electrophoresis for 16 h under 40 V (Left). Predicted RFLP patterns of BAC^PRV-G^ with *BamH* I, *Kpn* I, *Pst* I and *Sph* I digestion respectively. Predictions of these digestions were performed with the whole genome sequence of PRV ZJ01 strain (GenBank: KM061380.1) as reference. M: DL 15,000 DNA Marker (Takara)

Green plaques of mini-F recombinant PRV AH02LA were observed under UV light (488 nm), and isolated after several rounds of plaque purification. The mini-F recombinant PRV AH02LA DNA was isolated and transfected into *Escherichia coli* DH10B competent cells, the resulting clone was designated BAC^PRV-AH02LA^. BAC^PRV-AH02LA^ DNA was electroporated into *E. coli* GS1783 competent cells, resulting in a clone designated BAC^PRV-G^. This BAC was used for further gene manipulations using the *En Passant* protocol [20]. Restriction fragment length polymorphism (RFLP) analysis of BAC^PRV-G^ with *BamH* I, *Kpn* I, *Pst* I and *Sph* I generated patterns similar to those predicted from the PRV ZJ01 strain sequence (GenBank: KM061380.1) with minor differences (Fig. 2). Virus was rescued successfully by transfection of the BAC^PRV-G^ DNA into primary CECs, designated PRV AH02LA^R^. In summary, an infectious clone containing the complete genome of the PRV emerging variant AH02LA strain was constructed successfully.

### Construction of a gE deletion mutant virus

After co-transfection of BAC^PRV-G^ DNA with a DNA fragment containing the gE deletion (obtained through PCR), several white plaques were observed under UV illumination at 488 nm. One was isolated after several rounds of plaque purification and designated PRV LA-A^B^. The deletion was verified by PCR using primers of ΔgE TV overlap1 F andΔgE TV overlap2 R, followed by sequencing with the appropriate primers (Table 1).

### Growth kinetics of the PRV LA-A^B^ virus

Multistep growth kinetics for the PRV LA-A^B^, AH02LA, and AH02LA^R^ viruses were determined. Peak supernatant titers for LA-A^B^, AH02LA, and AH02LA^R^ were 10^8.18^, 10^9.01^, and 10^8.19^ TCID_50_ /mL respectively. At 48 hpi the titers for LA-A^B^ and AH02LA^R^ were not significantly different from AH02LA (*p* = 0.414 and 0.362). For cell-associated virus titers LA-A^B^, AH02LA, and AH02LA^R^ were 10^8.43^, 10^8.86^, and 10^8.13^ TCID_50_ /mL at 48 hpi, respectively. The titers of LA-A^B^ and AH02LA^R^ are not significant different from the parental virus AH02LA at 48 hpi (*p* = 0.126 and 0.088).

### Immunogenicity of the LA-A^B^ vaccine

**Fig. 3:**
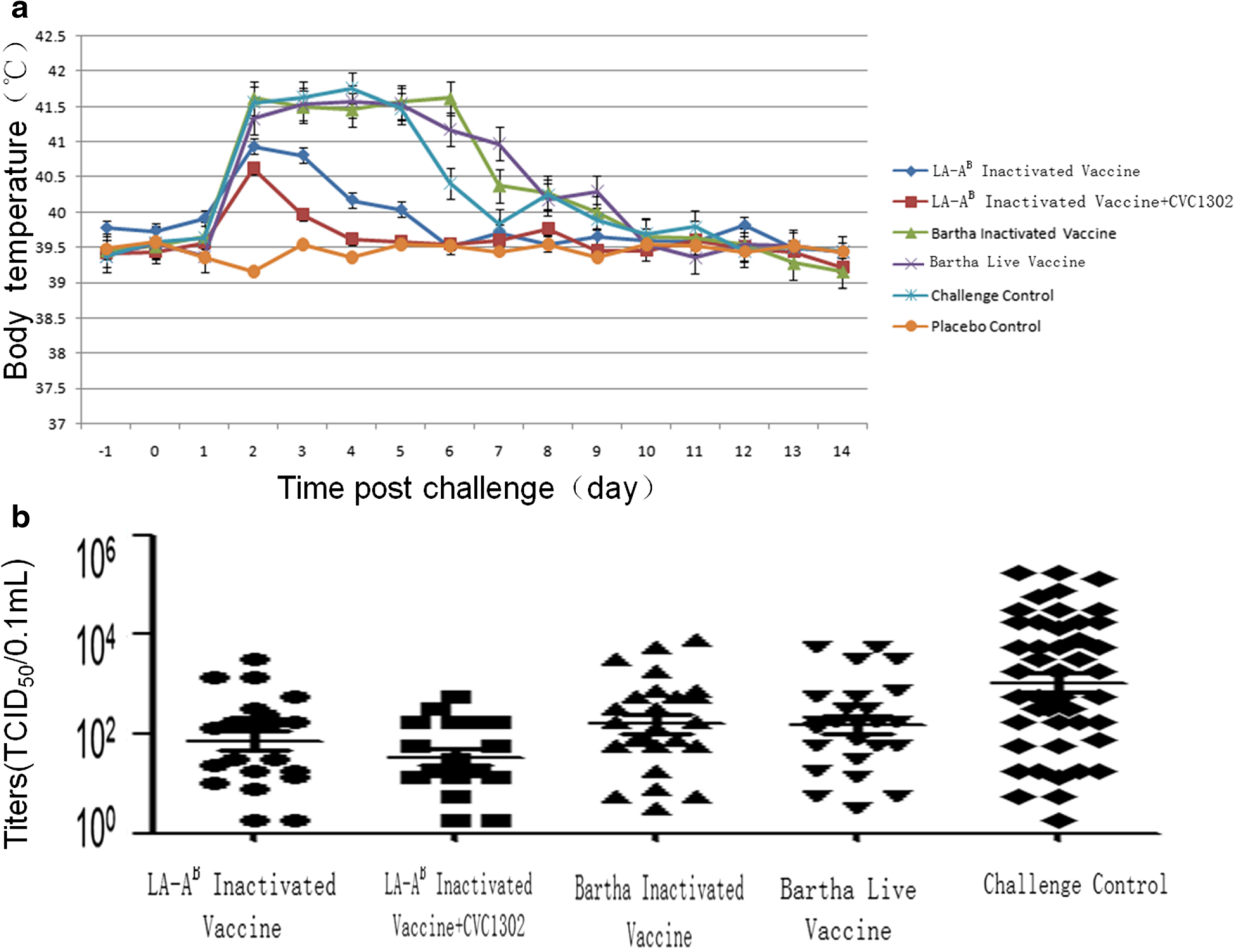
Body temperatures and virus shedding post challenge with PRV AH02LA strain. **a** Body temperatures of piglets in vaccinated groups were detected together with challenge control and placebo control from one day before challenge to 14 days post challenge. Average temperatures of five piglets of each group were taken for comparison. Error bars represent the standard deviations. **b** Nasal swab samples of all piglets of vaccinated groups together with challenge control and placebo control groups were collected before challenge and daily up to 14 days after challenge for virus isolation and titer determination. The titers of viruses were expressed as TCID_50_ on BHK-21 cells following the method of Karber. Average titers were determined with the samples that virus exceeding were detected

In challenge control group, all piglets had typical clinical syndromes of pseudorabies infection including sneezing, coughing, difficulty breathing and nasal discharge P.C.. Two piglets died at 7 days and two at 8 days P.C. (Table 2). Body temperatures of these piglets reached over 41 °C and lasted for 4–5 days (Fig. 3(a)). All the piglets shed virus with titers of 10^0.25^ ~ 10^5.25^ TCID_50_ /0.1 mL lasting for 5–7 days till death or 10 days for the surviving piglet (Fig. 3(b)). All piglets showed severe lung lesions from hemorrhaging and congestion (Table 2).

**Table 2:** Antibodies, clinical signs and lung lesions in animals

Groups			A	B	C	D	E	F
ELISA antibodies against PRV gB or gE	B.V.	gB+	0^a^/5^b^	0/5	0/5	0/5	0/5	0/5
		gE+	0/5	0/5	0/5	0/5	0/5	0/5
	7d P.V.	gB+	2/5	5/5	2/5	5/5	0/5	0/5
		gE+	0/5	0/5	0/5	0/5	0/5	0/5
	14d P.V.	gB+	5/5	5/5	5/5	5/5	0/5	0/5
		gE+	0/5	0/5	0/5	0/5	0/5	0/5
	21d P.V.	gB+	5/5	5/5	5/5	5/5	0/5	0/5
		gE+	0/5	0/5	0/5	0/5	0/5	0/5
	14d P.C.	gB+	5/5	5/5	5/5	5/5	2/2	0/5
		gE+	5/5	5/5	5/5	5/5	2/2	0/5
Mean titers of neutralizing antibodies	AH02LA	B.V.	<2^c^	<2	<2	<2	<2	<2
		7d P.V.	<2	<2	<2	<2	<2	<2
		14d P.V.	<2	0.4	<2	<2	<2	<2
		21d P.V.	2.00	2.80	<2	1.20	<2	<2
		28d P.V.	2.60	3.40	2.40	2.60	<2	<2
		35d P.V.	4.60	5.20	3.60	3.20	<2	<2
		42d P.V.	4.60	5.60	3.80	3.20	<2	<2
		49d P.V.	5.20	6.20	4.20	3.00	<2	<2
	Bartha K61	B.V.	<2	<2	<2	<2	<2	<2
		7d P.V.	<2	<2	<2	<2	<2	<2
		14d P.V.	<2	0.4	<2	<2	<2	<2
		21d P.V.	1.20	2.24	1.2	1.60	<2	<2
		28d P.V.	2.40	3.08	2.60	3.20	<2	<2
		35d P.V.	4.60	4.80	4.60	3.20	<2	<2
		42d P.V.	4.80	5.20	4.80	3.60	<2	<2
		49d P.V.	5.40	5.60	5.20	3.40	<2	<2
Clinical signs and lung lesions post challenge	Morbidity		1/5	0/5	4/5	3/5	5/5	0/5
	Duration of clinical signs(days)		1	-	2–4	2–3	4–7	-
	Mortality		0/5	0/5	0/5	0/5	4/5	0/5
	Lung lesions		0/5	0/5	2/5	1/5	5/5	0/5

*B.V* means before vaccination, *P.V* means post vaccination, *P.C* means post challenge. “a” indicates the number of piglets positive;“b” indicates the number of piglets in the group. “c” indicates mean titers of neutralizing antibody of each group expressed as nlog_2_ of highest dilution at which no CPE was observed

gB antibodies were detected in all piglets vaccinated with live Bartha K61 strain or the LA-A^B^ strain plus adjuvant at 7 days post vaccination(P.V.), while two of five piglets vaccinated LA-A^B^ strain alone or the inactivated Bartha K61 strain were anti-gB positive at 7 days P.V.. All samples collected at 14 days P.V. were positive for gB antibodies (Table 2). This result indicates the adjuvant CVC1302 helps the LA-A^B^ vaccine stimulate immunity against PRV more quickly. At 14 days P.C. all piglets were positive for gE antibodies (Table 2).

No neutralizing antibodies (NA) against the AH02LA or Bartha K61 strain, were detected in any of the piglets at 7 days P.V.. At 14 days P.V. neutralizing antibodies were detected in one piglet from group B (LA-A^B^ plus adjuvant) at a titer of 2. In groups A, B, and C, NA titers began increasing quickly seven days after the boost and continued to increase over the next two weeks. The LA-A^B^ vaccines induced higher titers of NA against the AH02LA and Bartha K61 strain, than vaccines made of Bartha K61. One dose of live Bartha K61 vaccine stimulated a weak NA titer of less than 4 against the AH02LA strain or the Bartha K61 strain. These results indicate that optimizing the prime-boost regimen is necessary for live Bartha K61 vaccine to stimulate strong humoral immunity. NA titers in group B piglets were higher than other groups, indicating that adjuvant CVC1302 helped stimulate higher specific immunity for LA-A^B^ vaccine (Table 2).

All piglets in the vaccinated groups (A, B, C and D) were protected against lethal challenge. Fevers in group A and B piglets were transient, and other clinical symptoms were mild or absent respectively. Titers of shed virus in LA-A^B^ vaccinated group A piglets were 10^0.25^ ~ 10^3.75^ TCID_50_/0.1 mL lasting 3–4 days; in LA-A^B^ plus adjuvant group B piglets, titers were 10^0.125^ ~ 10^2.875^ TCID_50_/0.1 mL lasting 3–4 days. Piglets in groups C and D (vaccinated with inactivated Bartha K61 and live Bartha K61 respectively) presented fevers of more than 41 °C lasting for 4–6 days (Fig. 3(a)), four and three piglets respectively, presented sneezing lasting for 2–4 days and nasal discharge lasting 2–3 days (Table 2). The titers of shed virus in these piglets were 10^0.125^ ~ 10^4.125^ TCID_50_ /0.1 mL lasting for 5–6 days (Fig. 3(b)). No Lung lesions were observed in any group A or B piglets, while 2/5 group C piglets and in 1/5 group D piglets presented lesions from hemorrhage and congestion (Table 2).

## Discussion

The results of vaccination test demonstrate that vaccines made of PRV LA-A^B^ elicit a more effective immune response against the emergent PRV AH02LA than the Bartha K61 vaccines. The immune response is even more pronounced when PRV LA-A^B^ is administered with adjuvant. Pigs vaccinated with LA-A^B^ produced lower titers of virus shedding over a shorter duration than pigs with Bartha K61. This is a clear advantage in the terms of controlling the spread of infection. Adjuvant CVC1302 is a mixture of monophosphoryl lipid A (MPLA), muramyl dipeptide (MDP) and β-glucan. This mixture has been shown to improve the efficiency of the inactivated vaccines of porcine epidemic diarrhea virus and foot and mouth disease virus (Patent:CN103083663B). MPLA, MDP and β-glucan can serve as delivery systems or stimulators for the immune system [28–31].

Bacterial artificial chromosome(BAC) of herpesvirus is a useful tool for generation of gene modified mutants to study the mechanism of the viruses. After the construction of the first BAC of mouse cytomegalovirus (MCMV) [32], a few of herpesvirus genomes have been maintained in BACs as infectious clones, which have provided great help for discovery of pathology of these viruses [27, 33] and construction of vectored vaccines [26, 34, 35], especially following the protocol of En Passant protocol [23]. The parental PRV AH02LA strain of the BAC constructed in this study is a virulent variant strain that caused severe losses in many porcine farms in China. So even though En Passant protocol was not performed in the generation of the gE deleted LA-A^B^ strain in this study, it will be used for further analysis of the mechanism of the high virulence of this variant.

The latent infection of PRV is always a potential threat to porcine herds [36]. So the eradication of PRV has been a direct aim of many farms for control of this disease. This aim has been achieved in a few countries [1] and the infection of wild PRV has been controlled to very low levels in some individual farms in China before 2011 [37, 38]. DIVA vaccine(vaccine allowing differentiating infected from vaccinated animals) has played an important role towards this aim [1]. In the project of eradication of PRV, the efficient reduction of virus shedding post infection with virulent strain is crucial [2]. In this study, the inactivated vaccine of LA-A^B^ strain with adjuvant CVC1302 reduced virus shedding significantly, despite that all vaccinated animals did not stop virus shedding post challenge.

We did find a difference in the profile of clinical symptoms between animals challenged through I.M. or I.N. route. During the challenge test in this study, we also tried the challenge route of I.M. injection for 11-week-old piglets (data not shown). We found that even though it caused high fever and obvious clinical syndromes of PR through this way, the virulence was significantly lower than through I.N. way. This difference is presumed to be associated with the increased titers of virus after replication on epithelial cells of respiratory tract. To mimick the nature infection of domestic pigs genuinely, we did the challenge protection test through I.N. way for evaluation of the vaccines in this study.

## Conclusion

The infectious clone consists of the whole genome of PRV emerging variant AH02LA strain. Inactivated vaccine made of PRV LA-A^B^ strain can provide better clinical protection than vaccines made of Bartha K61. In addition, significant decrease of virus shedding was observed post lethal challenge when LA-A^B^ inactivated vaccine were adjuvanted with CVC1302.

## Abbreviations

BAC: Bacterial artificial chromosome
CEC: Chicken embryo cell
CPE: Cytopathogenic effects
*E.coli*: Escherichia coli
ELISA: Enzyme-linked immunosorbent assay
gB: glycoprotein B
gE: glycoprotein E
gI: glycoprotein I
Hpi: Hour post infection
I.M: Intramuscularly
I.N: Intranasally
LD_50_: 50% lethal dose
MCMV: Mouse cytomegalovirus
MDP: Muramyl dipeptide
MOI: Multiplicity of infection
MPLA: Monophosphoryl lipid
NA: Neutralizing antibodies
ORF: Open reading frame
PC: Post challenge
PV: Post vaccination
PBS: Phosphate buffer saline
PCR: Polymerase chain reaction
PRV: Pseudorabies virus
RFLP: Restriction fragment length polymorphism
TCID_50_: 50% tissue culture infectious dose
TK: Thymidine kinase
VN: Virus neutralization
